# Prevalence and risk factors of burnout among community pharmacists in Saudi Arabia: a cross-sectional study

**DOI:** 10.3389/fpubh.2025.1706598

**Published:** 2025-12-12

**Authors:** Abdulrahman M. Hijri, Fahad Alzahrani, Osama A. Madkhali, Majid Ali, Mazen A. Alaqil, Riyadh M. Salami, Yaseen A. Madkhali, Abdulrahman A. Almalki, Emad H. Hassany, Asma M. Ahmed, Khalid H. Khubrani, Nasser M. Alsubaie, Aseel A. Alsraeya

**Affiliations:** 1 Department of Pharmaceutical Care Services, King Abdulaziz Medical City, Riyadh, Saudi Arabia; 2 Department of Pharmacy Practice, College of Pharmacy, Taibah University, Madinah, Saudi Arabia; 3 Department of Pharmaceutics, Collage of Pharmacy, Jazan University, Jazan, Saudi Arabia; 4 Department of Basic Sciences, College of Medicine, Sulaiman Alrajhi University, Al-Bukayriyah, Saudi Arabia; 5 Pharmacy Practice Research Unit, College of Pharmacy, Jazan University, Jazan, Saudi Arabia; 6 Pharmaceutical Care Services, Prince Sultan Cardiac Center, Riyadh, Saudi Arabia; 7 Al-Dawaa Medical Services Company, Riyadh, Saudi Arabia; 8 Al-Dawaa Medical Services Company, Jazan, Saudi Arabia; 9 Pharmaceutical Care Administration, Armed Forces Hospital Southern Region, Khamis Mushait, Saudi Arabia

**Keywords:** burnout, stress, wellbeing, community pharmacists, workforce, Saudi Arabia, occupational health

## Abstract

**Background:**

Burnout among healthcare professionals impacts patient care quality and healthcare systems. While extensively studied across various healthcare sectors, burnout among community pharmacists in Saudi Arabia remains underexplored despite their expanding roles in healthcare delivery.

**Methods:**

This cross-sectional study utilized an online survey with the validated Maslach Burnout Inventory-Human Services Survey (MBI-HSS) to assess burnout across three dimensions among community pharmacists in Saudi Arabia from December 2024 to March 2025. Multiple regression analysis identified factors associated with burnout.

**Results:**

Among 408 community pharmacists (mean age 28.07 ± 3.75 years, 72.8% male), high levels of burnout were prevalent: 65.9% exhibited high emotional exhaustion (EE) (mean 35.50 ± 14.40), 64.5% showed high depersonalization (DP) (mean 14.66 ± 8.26), and 66.2% demonstrated low personal accomplishment (mean 29.34 ± 11.16). Sales pressure frequency was the strongest predictor of EE (*β* = 0.312, *p* < 0.001) and a significant predictor of DP (*β* = 0.218, *p* < 0.001). Saudi nationality was strongly associated with both EE (*β* = 0.267, *p* < 0.001) and DP (*β* = 0.298, *p* < 0.001). Years of experience showed protective effects against burnout dimensions. Pharmacists experiencing constant sales pressure had 4.23 times higher odds of high EE (95% CI: 2.18–8.21, *p* < 0.001).

**Conclusion:**

Community pharmacists in Saudi Arabia experience alarmingly high burnout levels, particularly among Saudi nationals, those facing constant sales pressure, practitioners with less experience, and those managing high patient volumes. These findings highlight the urgent need for workplace interventions, policy changes, and support systems focusing on reducing commercial pressures, optimizing workload distribution, and providing targeted support for vulnerable subgroups.

## 1 Introduction

Burnout syndrome has emerged as a critical occupational health concern across healthcare professions worldwide, characterized by emotional exhaustion, depersonalization, and reduced personal accomplishment resulting from chronic workplace stress (1). Originally conceptualized by Freudenberger in 1974 and later operationalized through the Maslach Burnout Inventory, burnout represents a psychological syndrome that develops in response to chronic interpersonal stressors in the workplace (2). The healthcare sector, with its inherent demands of patient care, high-stakes decision-making, and emotional labor, has been identified as particularly susceptible to burnout-related challenges.

The prevalence of burnout among healthcare professionals has reached alarming levels globally, with pharmacists across various practice settings demonstrating particularly concerning rates. A comprehensive systematic review by Dee et al. highlighted a significant prevalence of burnout in pharmacists, indicating a pressing issue that warrants urgent investigation (3). The review indicated that burnout among community pharmacists can manifest in several forms, including personal burnout, work-related burnout, and client-related burnout, with studies utilizing specialized burnout measurement instruments reporting prevalence rates of approximately 56.7 to 89.7% among community pharmacists (3). International studies have consistently documented high burnout rates across different regions. A French nationwide cross-sectional study reported burnout rates approaching 56.2% (with 10.5% of them presented with severe burnout syndrome) in a sizable cohort of community pharmacists, underscoring the universal nature of this concern (4). These figures align with findings from other developed countries, where burnout not only impacts the well-being of pharmacists but also threatens patient safety and the quality of pharmaceutical care provided (5).

Community pharmacy practice has undergone significant transformation over the past decades, evolving from a traditional dispensing-focused model to a comprehensive pharmaceutical care approach emphasizing patient counseling, medication therapy management, and clinical services (6). Community pharmacists, who often serve as the most accessible healthcare professionals, are increasingly burdened by factors such as high workload, consumer demands, and an evolving healthcare landscape, potentially exacerbating their risk for burnout (7, 8). Several factors contribute to the development of burnout among community pharmacists, with research indicating that workplace characteristics, rather than individual demographics, play a more significant role in determining burnout levels. A study in Turkey emphasized that organizational factors were more substantial contributors to burnout than personal characteristics (9). This viewpoint aligns with research highlighting organizational factors such as inadequate administrative support and increased non-clinical duties as substantial contributors to burnout (10, 11). The consequences of burnout extend beyond the individual, influencing job satisfaction and retention rates among pharmacists. Research illustrates that a substantial percentage of pharmacists express a desire to leave the profession, with turnover intentions reported at alarming levels. This potential exodus poses significant challenges to the healthcare system, as persistent turnover can lead to disruptions in patient care and ultimately compromise public health outcomes (12, 13).

Saudi Arabia’s healthcare system has undergone substantial transformation as part of the Vision 2030 initiative, emphasizing healthcare quality improvement, accessibility enhancement, and professional development (14). The pharmaceutical sector has experienced significant growth, with expanding community pharmacy networks and evolving practice standards. The Saudi Food and Drug Authority has implemented comprehensive regulations governing pharmacy practice, emphasizing patient safety and the quality of pharmaceutical care (15). The demographic profile of Saudi Arabia, characterized by a young population with increasing prevalence of chronic diseases such as diabetes and cardiovascular conditions, presents unique challenges for community pharmacists (16). Cultural factors, including patient expectations, family involvement in healthcare decisions, and preferences for traditional medicine, add complexity to pharmaceutical care delivery (17). Understanding burnout prevalence and associated risk factors is essential for developing targeted interventions, informing policy decisions, and supporting pharmacist well-being within the Saudi healthcare context. Furthermore, the rapid evolution of pharmacy practice in Saudi Arabia, coupled with increasing patient care responsibilities and regulatory requirements, may create unique stressors that warrant systematic investigation (18, 19). Nevertheless, the evidence remains limited regarding the broader cultural, regional, and occupational factors influencing burnout among pharmacists in Saudi Arabia. Therefore, there is a need for a comprehensive assessment that reflects the current post-pandemic context and diverse work environments of Saudi pharmacists. This study addresses these gaps by providing updated insights that can guide targeted well-being strategies and inform national workforce policies. Therefore, this study aimed to assess the prevalence and associated factors of burnout among pharmacists in Saudi Arabia.

## 2 Materials and methods

### 2.1 Study design and setting

This study employed a cross-sectional survey design to investigate the prevalence and risk factors of burnout among community pharmacists in Saudi Arabia. The study was conducted across all five regions of Saudi Arabia (Central, Western, Southern, Eastern, and Northern provinces) from December 2024 to March 2025 to ensure geographical representation and generalizability of findings. Pharmacists licensed to practice in Saudi Arabia who had a bachelor’s pharmacy degree or a higher pharmacy education degree and are willing to complete the questionnaire, were eligible to participate in this study.

### 2.2 Participants and sampling

The target population comprised licensed community pharmacists working in both chain and independent pharmacies across Saudi Arabia. Participants were eligible for inclusion if they were: (1) licensed pharmacists working in community pharmacy settings, (2) currently employed in Saudi Arabia, and (3) willing to provide informed consent. Pharmacists working exclusively in hospital or clinical settings were excluded from the study. A convenience sampling method was employed to recruit participants through professional networks and social media platforms, including X and WhatsApp groups dedicated to pharmacy professionals in Saudi Arabia.

### 2.3 Data collection instrument

The questionnaire comprised two main sections: Section I: Sociodemographic and Work-Related Characteristics, and Section II: Burnout Assessment - Maslach Burnout Inventory-Human Services Survey (MBI-HSS).

Section I captured sociodemographic and work-related variables: gender, age, nationality, marital status, pharmacy and current residence locations, nationality, marital status, income, educational level, years of experience, working hours, number of patients served, number of trainees at the site, night shift frequency, and number of other staff in the pharmacy. Selection of these variables was informed by prior pharmacist burnout literature (3, 9–13).

Burnout was assessed in Section II using the Maslach Burnout Inventory-Human Services Survey (MBI-HSS), the gold standard instrument for measuring burnout among human services professionals (2). The license to use this instrument was purchased from Mind Garden (20). The MBI-HSS was developed by Maslach and Jackson (2) and has been extensively validated across diverse healthcare settings and cultural contexts (21–26). The MBI-HSS consists of 22 items that assess three distinct dimensions (subscales) of burnout (1) Emotional Exhaustion (EE) - 9 items, (2) Depersonalization (DP) - 5 items, and (3) Personal Accomplishment (PA) - 8 items. Each item is rated on a 7-point Likert scale indicating frequency of experience: 0 = Never, 1 = A few times a year or less, 2 = Once a month or less, 3 = A few times a month, 4 = Once a week, 5 = A few times a week, and 6 = Every day.

Subscale Score Calculation was as follows: Emotional Exhaustion (EE): Sum of items 1, 2, 3, 6, 8, 13, 14, 16, 20 (Range: 0–54), Depersonalization (DP): Sum of items 5, 10, 11, 15, 22 (Range: 0–30), and Personal Accomplishment (PA): Sum of items 4, 7, 9, 12, 17, 18, 19, 21 (Range: 0–48). Burnout Level Classify into: Emotional Exhaustion: Low (≤20), Moderate (21–30), High (≥31), Depersonalization: Low (≤5), Moderate (6–10), High (≥11), and Personal Accomplishment: Low burnout (≥42), Moderate (36–41), High burnout (≤35).

Note: For Personal Accomplishment, lower scores indicate higher burnout, as this dimension is inversely related to burnout compared to the other two dimensions.

Face/content adequacy of the questionnaire was confirmed by two academic pharmacy practice experts and one occupational health specialist (minor wording refinements; no items removed). The final instrument was administered in English.

### 2.4 Sample size calculation

The sample size was calculated using the formula for cross-sectional studies investigating prevalence *n* = Z^2^α/2 × P(1-P)/d^2^ where Z^2^α/2 = 1.96^2^ (for 95% confidence interval), *P* = expected prevalence of burnout, and *d* = margin of error (5%).

The total number of community pharmacists working in Saudi Arabia in 2023 is approximately 20,900 (27). Based on previous studies examining burnout among healthcare professionals in the Middle East region, an estimated prevalence of 50% was used to ensure maximum sample size (28, 29). Using a 95% confidence interval and a 5% margin of error, the calculated minimum sample size was 384 participants. To account for potential non-response and incomplete surveys, the target sample size was increased by 10%, resulting in a target of 422 participants. The final sample included 408 participants, which exceeded the minimum required sample size and provided adequate statistical power (>80%) for the planned analyses (30).

### 2.5 Data collection

Data were collected using a self-administered, anonymous online questionnaire developed using Google Forms. The questionnaire was distributed through various channels, including Direct sharing through professional pharmacy networks, social media platforms (X and WhatsApp), and Email distribution to pharmacy chains and independent pharmacies.

### 2.6 Statistical analysis

Data analysis was conducted using SPSS version 28.0 (IBM Corporation, Armonk, NY, USA). Descriptive statistics, including means, standard deviations, frequencies, and percentages, were calculated for all variables. Normality of continuous variables was assessed using the Kolmogorov–Smirnov test.

For bivariate analyses, independent t-tests were used to compare burnout scores between two groups for normally distributed data. Mann–Whitney U tests were employed for non-normally distributed continuous variables. One-way ANOVA was used to compare burnout scores across multiple groups. Chi-square tests were used for categorical variables.

### 2.7 Ethical considerations

Ethical approval for this study was obtained from the Local Committee for Research Ethics at Jazan University (Approval Number: HAPO-10-Z-001). Informed consent was obtained implicitly through participants’ voluntary completion and submission of the survey, as clearly stated at the beginning of the questionnaire. Prior to survey commencement, all participants were presented with comprehensive information to facilitate informed decision-making regarding their participation. All data were collected anonymously with no personally identifying information recorded. Data were stored securely on password-protected systems with access limited to authorized research team members.

## 3 Results

### 3.1 Sociodemographic data

A total of 408 community pharmacists participated in this cross-sectional study conducted across all five provinces of Saudi Arabia. Key sociodemographic and work-related characteristics are presented in Tables 1, 2.

**Table 1:** Sociodemographic characteristics with MBI subscale scores (*n* = 408).

Characteristic	*n* (%)	EE Mean	DP Mean	PA Mean
Age (years)				
Mean ± SD	28.07 ± 3.75	35.50	14.66	29.34
Range	22–47	0–54	0–30	0–48
Gender				
Male	297 (72.8)	34.68	14.95	29.38
Female	111 (27.2)	37.70	13.86	29.23
P value^1^		*p* > 0.05	*p* > 0.05	*p* > 0.05
Nationality				
Saudi	347 (85.0)	37.27	15.72	29.19
Non-Saudi	61 (15.0)	25.44	8.59	30.20
*P* value^1^		*p* < 0.001*	*p* < 0.001*	*p* > 0.05
Marital status				
Unmarried/Single	286 (70.1)	36.92	15.13	29.43
Married	115 (28.2)	31.95	13.34	29.19
Divorced/Widowed	7 (1.7)	36.14	17.00	28.29
*P* value^2^		*p* < 0.05*	*p* > 0.05	*p* > 0.05
Province				
Southern	149 (36.5)	35.31	14.52	29.65
Central	125 (30.6)	33.77	13.58	28.64
Western	60 (14.7)	40.73	16.90	30.45
Eastern	46 (11.3)	37.30	15.63	29.78
Northern	28 (6.9)	36.25	14.92	28.46
*P* value^2^		*p* < 0.01*	*p* > 0.05	*p* > 0.05
Monthly income (SAR)				
5,000-10,000	287 (70.3)	35.79	14.39	29.93
11,000-15,000	89 (21.8)	36.76	16.16	28.66
< 5,000	23 (5.6)	27.22	11.00	24.30
> 15,000	9 (2.2)	35.00	17.78	30.00
*P* value^2^		*p* < 0.05*	*p* < 0.05*	*p* < 0.05*
Education level				
PharmD	250 (61.3)	37.66	15.86	29.51
Bachelor’s (BPharm)	151 (37.0)	32.15	12.75	29.02
Master’s	7 (1.7)	30.71	12.86	30.29
*P* value^2^		*p* < 0.01*	*p* < 0.01*	*p* > 0.05
Location of residence				
Urban	337 (82.6)	35.81	14.90	29.43
Rural	71 (17.4)	34.04	13.49	28.92
*P* value^1^		*p* > 0.05	*p* > 0.05	*p* > 0.05
Years of experience				
1–5 years	244 (59.8)	37.72	16.04	29.58
< 1 year	95 (23.3)	35.18	13.38	28.58
6–10 years	43 (10.5)	29.16	12.49	29.67
11–15 years	16 (3.9)	29.75	10.94	29.25
> 15 years	10 (2.5)	21.10	8.40	29.40
*P* value^2^		*p* < 0.001*	*p* < 0.001*	*p* > 0.05

EE, emotional exhaustion; DP, depersonalization; PA, personal accomplishment.

*Statistically significant.

^1^Independent *t*-test.

^2^ANOVA.

**Table 2:** Work-related characteristics with MBI subscale scores (*n* = 408).

Characteristic	*n* (%)	EE Mean	DP Mean	PA Mean
Working days per week				
5 days	38 (9.3)	33.26	14.74	30.03
6 days	332 (81.4)	36.53	15.31	29.19
7 days	38 (9.3)	28.82	8.84	30.00
*P* value^2^		*p* < 0.01*	*p* < 0.001*	*p* > 0.05
Working hours per day				
<8 h	10 (2.5)	20.80	8.00	20.80
8 h	152 (37.3)	33.12	13.70	28.97
9 h	176 (43.1)	38.93	16.20	29.93
10 h	43 (10.5)	38.00	16.19	29.74
11 h	6 (1.5)	26.50	11.00	23.67
≥12 h	21 (5.1)	28.57	9.67	31.95
*P* value^2^		*p* < 0.001*	*p* < 0.001*	*p* < 0.05*
Pharmacy type				
Chain pharmacy	357 (87.5)	36.13	14.91	29.75
Independent pharmacy	51 (12.5)	31.10	12.88	26.51
*P* value^1^		*p* < 0.05*	*p* > 0.05	*p* > 0.05
Number of pharmacists				
1–2	170 (41.7)	35.13	13.97	29.14
3–4	186 (45.6)	36.27	15.09	29.34
5–6	34 (8.3)	34.26	14.97	31.06
> 6	18 (4.4)	33.44	16.06	28.00
*P* value^2^		*p* > 0.05	*p* > 0.05	*p* > 0.05
Patients served per day				
10–30	42 (10.3)	29.95	10.19	26.64
31–60	65 (15.9)	32.46	13.65	29.74
61–100	86 (21.1)	36.90	14.97	30.55
>100	215 (52.7)	36.95	15.71	29.27
*P* value^2^		*p* < 0.01*	*p* < 0.001*	*p* > 0.05
Students trained (Last 6 months)				
None	214 (52.4)	36.57	14.66	29.34
1–2	132 (32.4)	34.93	14.97	29.35
≥3	62 (15.2)	33.05	13.97	29.32
*P* value^2^		*p* > 0.05	*p* > 0.05	*p* > 0.05
Night shift assignment				
Yes	357 (87.5)	35.59	14.91	29.36
No	51 (12.5)	34.88	12.90	29.20
*P* value^1^		*p* > 0.05	*p* > 0.05	*p* > 0.05
Sales pressure frequency				
Always	208 (51.0)	40.53	16.92	29.21
Often	76 (18.6)	34.86	14.30	31.30
Sometimes	88 (21.6)	28.74	11.61	28.26
Rarely	18 (4.4)	22.44	9.06	29.11
Never	18 (4.4)	26.28	10.50	28.06
*P* value^2^		*p* < 0.001*	*p* < 0.001*	*p* > 0.05
Number of pharmacy assistants				
0	150 (36.8)	35.16	14.67	28.92
1	149 (36.5)	35.37	14.48	29.76
2–4	98 (24.0)	36.76	15.00	30.14
≥5	11 (2.7)	26.64	12.18	26.45
*P* value^2^		*p* > 0.05	*p* > 0.05	*p* > 0.05
Profession change likelihood (Next 5 years)				
Very likely	169 (41.4)	40.27	16.09	29.63
Somewhat likely	87 (21.3)	33.99	12.56	30.23
Neither likely nor unlikely	70 (17.2)	30.67	13.24	29.06
Somewhat unlikely	37 (9.1)	29.43	14.00	27.00
Very unlikely	45 (11.0)	33.07	16.04	28.89
*P* value^2^		*p* < 0.001*	*p* < 0.05*	*p* > 0.05

EE, emotional exhaustion; DP, depersonalization; PA, personal accomplishment.

*Statistically significant.

^1^Independent *t*-test.

^2^ANOVA.

### 3.2 Prevalence of burnout

**Figure 1:**
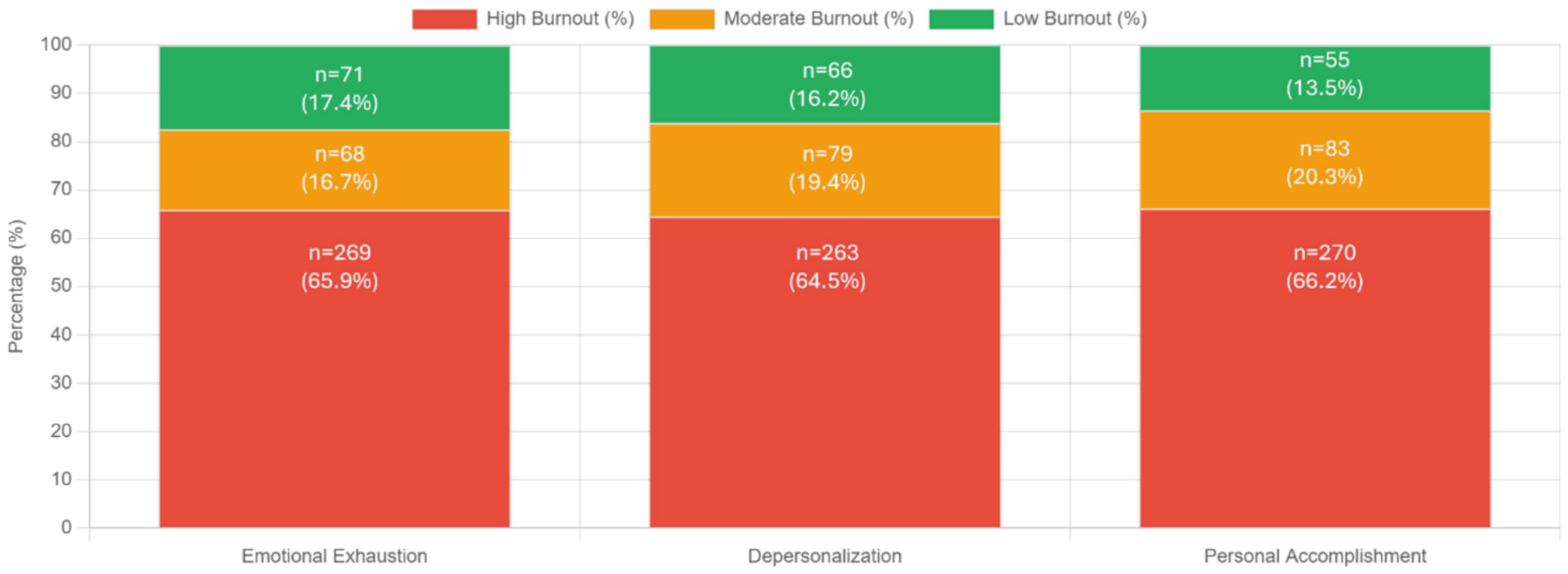
Burnout prevalence across MBI dimensions.

The prevalence of burnout among community pharmacists, as measured by the MBI-HSS, revealed alarmingly high rates across all three dimensions (Table 3; Figure 1). The mean scores for each burnout dimension were: EE 35.50 ± 14.40, DP 14.66 ± 8.26, and PA 29.34 ± 11.16. For EE, 269 participants (65.9%) exhibited high levels, 68 (16.7%) showed moderate levels, and only 71 (17.4%) demonstrated low levels. DP patterns were similarly concerning, with 263 participants (64.5%) showing high levels, 79 (19.4%) moderate levels, and 66 (16.2%) low levels. PA, which is inversely related to burnout (lower scores indicate higher burnout), showed that 270 participants (66.2%) had high burnout (low personal accomplishment), 83 (20.3%) had moderate levels, and only 55 (13.5%) showed low burnout (high personal accomplishment).

**Table 3:** Maslach burnout inventory scores and burnout prevalence (*n* = 408).

MBI dimension	Mean ± SD	Burnout level	*n* (%)
Emotional exhaustion (0–54)	35.50 ± 14.40	High (≥31)	269 (65.9)
		Moderate (21–30)	68 (16.7)
		Low (≤20)	71 (17.4)
Depersonalization (0–30)	14.66 ± 8.26	High (≥11)	263 (64.4)
		Moderate (6–10)	79 (19.4)
		Low (≤5)	66 (16.2)
Personal accomplishment (0–48)	29.34 ± 11.16	High burnout (≤35)	270 (66.2)
		Moderate (36–41)	83 (20.3)
		Low burnout (≥42)	55 (13.5)

These findings indicate that approximately two-thirds of community pharmacists in Saudi Arabia are experiencing high levels of burnout across all three measured dimensions. The consistency of high burnout rates across EE (65.9%), DP (64.5%), and PA (66.2%) suggests a pervasive burnout crisis within the community pharmacy profession in Saudi Arabia.

### 3.3 Factors associated with burnout: multivariable analysis

**Figure 2:**
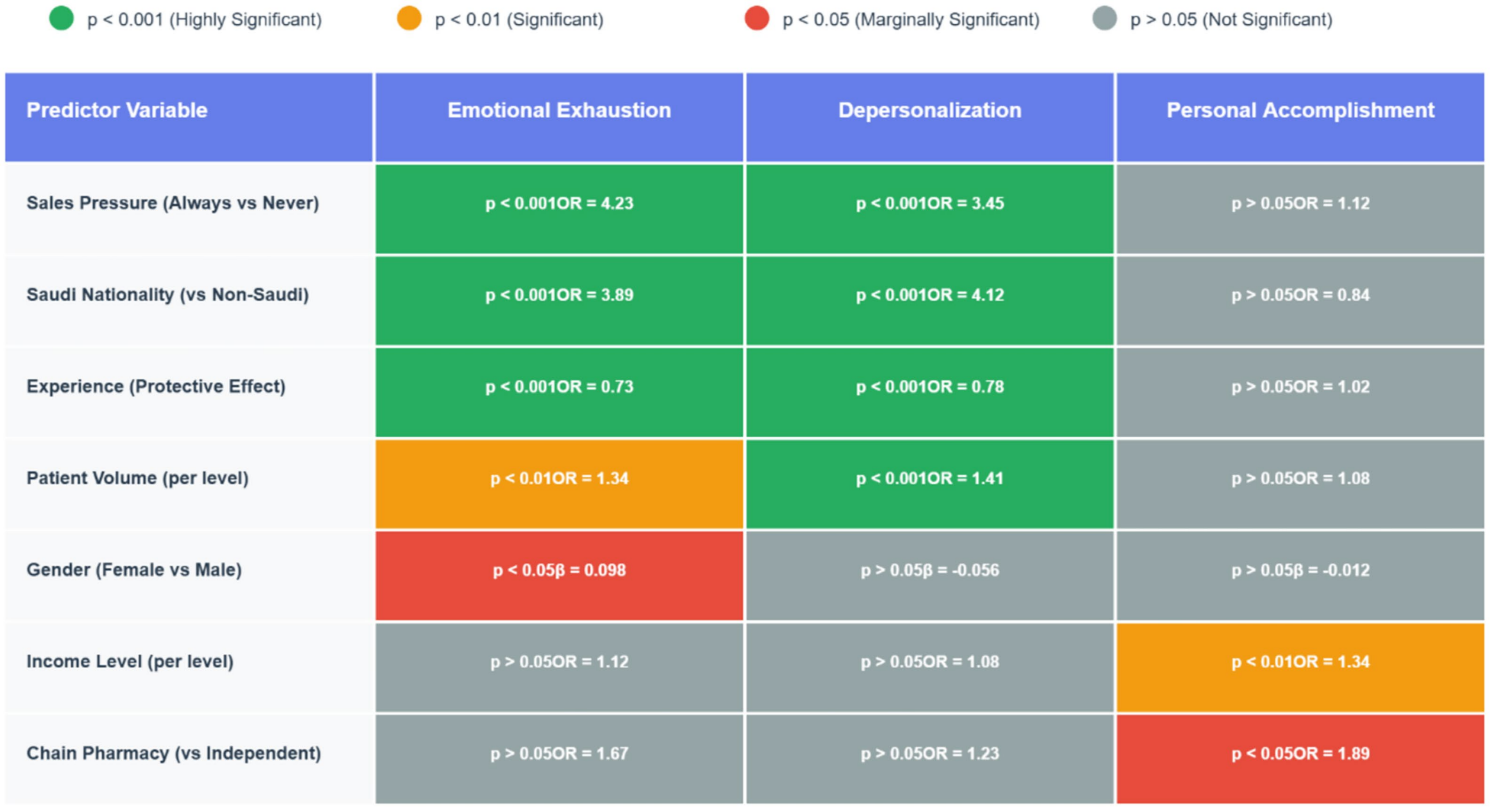
Significance matrix: which factors predict which burnout dimensions. Heat map table showing statistical significance levels and effect sizes (OR = Odds Ratio, *β* = Beta coefficient) for each predictor across the three burnout dimensions. Key Finding: notice how Personal Accomplishment (rightmost column) has a completely different pattern - only Income and Chain Pharmacy are significant, while traditional stressors (Sales Pressure, Nationality, Experience) show no effect.

Multiple linear regression analysis was conducted to identify independent predictors of all three burnout dimensions while controlling for potential confounding variables (Table 4; Figure 2). The models included age, gender, nationality, sales pressure frequency, years of experience, patient volume, pharmacy type, and night shift assignment as predictors. The regression models demonstrated dramatically different explanatory power across dimensions: good explanatory power for emotional exhaustion [R^2^ = 0.284, *F*(8,399) = 19.78, *p* < 0.001], moderate explanatory power for depersonalization [R^2^ = 0.198, F(8,399) = 12.34, *p* < 0.001], and weak explanatory power for personal accomplishment [R^2^ = 0.045, F(8,399) = 2.35, *p* < 0.05].

**Table 4:** Multiple linear regression results for burnout dimensions.

Predictor variable	Emotional exhaustion (EE)	Depersonalization (DP)	Personal accomplishment (PA)
Model statistics			
R^2^ (R-squared)	0.284***	0.198***	0.045*
Adjusted R^2^	0.270	0.182	0.026
F-statistic	F(8,399) = 19.78***	F(8,399) = 12.34***	F(8,399) = 2.35*
Sample size (*n*)	408	408	408
Standardized coefficients (*β*)			
Age	*β* = −0.089*	*β* = −0.067	*β* = 0.043
Gender (Female = 1)	*β* = 0.098*	*β* = −0.056	*β* = −0.012
Saudi national (Yes = 1)	*β* = 0.267***	*β* = 0.298***	*β* = −0.071
Sales pressure frequency	*β* = 0.312***	*β* = 0.218***	*β* = 0.023
Years of experience	*β* = −0.134**	*β* = −0.089*	*β* = 0.067
Patient volume per day	*β* = 0.089*	*β* = 0.123**	*β* = 0.034
Chain pharmacy (Yes = 1)	*β* = 0.076	*β* = 0.054	*β* = 0.087
Night shift (Yes = 1)	*β* = 0.034	*β* = 0.067	*β* = 0.012

***p < 0.001; **p < 0.01, *p < 0.05. Note the dramatically different model performance: EE and DP models explain substantial variance while the PA model explains minimal variance, indicating different underlying mechanisms.

Sales pressure frequency emerged as the strongest independent predictor of EE (*β* = 0.312, *p* < 0.001) and a significant predictor of DP (*β* = 0.218, *p* < 0.001), but showed no significant association with PA (*β* = 0.023, *p* > 0.05). Saudi nationality remained a highly significant independent predictor for both EE (*β* = 0.267, *p* < 0.001) and DP (*β* = 0.298, *p* < 0.001), but not for PA (*β* = −0.071, *p* > 0.05), even after controlling for other variables.

Years of experience showed significant protective effects against EE (*β* = −0.134, *p* < 0.01) and DP (*β* = −0.089, *p* < 0.05), but no significant association with PA (*β* = 0.067, *p* > 0.05), confirming that experience protects against stress-related burnout but does not enhance sense of accomplishment. Female gender emerged as a significant independent predictor of EE in the multivariable model (*β* = 0.098, *p* < 0.05), but showed no significant associations with DP (*β* = −0.056, *p* > 0.05) or PA (*β* = −0.012, *p* > 0.05).

Patient volume per day showed significant positive associations with both EE (*β* = 0.089, *p* < 0.05) and DP (*β* = 0.123, *p* < 0.01), but no significant association with PA (*β* = 0.034, *p* > 0.05), supporting the hypothesis that higher workload contributes to stress and cynicism but not necessarily to reduced sense of accomplishment.

## 4 Discussion

This cross-sectional study investigated the prevalence and risk factors of burnout among community pharmacists in Saudi Arabia, revealing concerning levels of burnout across all three dimensions measured by the Maslach Burnout Inventory. The findings indicate that approximately 66% of community pharmacists experience high emotional exhaustion, 64.5% report high depersonalization, and 66.2% demonstrate reduced personal accomplishment. Our analysis identified several significantly associated risk factors, with sales pressure emerging as the strongest predictor (OR = 4.23 for emotional exhaustion), followed by Saudi nationality (OR = 3.89 for emotional exhaustion), high patient volume (OR = 1.34 for emotional exhaustion), and limited professional experience showing protective effects (OR = 0.73 for emotional exhaustion). The high prevalence observed underscores the significant occupational health challenge facing the pharmacy profession in Saudi Arabia and highlights the urgent need for targeted interventions.

The high prevalence of burnout observed across all three dimensions exceeds rates reported in international studies. The French nationwide BOP (Burnout among Pharmacist) study by Balayssac et al. (4) found a burnout prevalence of 56.2% among community pharmacists, while Jones et al. (31) reported burnout rates of approximately 53.2% among hospital clinical pharmacists in the United States. Our findings of 65.9% high emotional exhaustion, 64.5% high depersonalization, and 66.2% reduced personal accomplishment suggest that community pharmacists in Saudi Arabia face particularly intense workplace stressors compared to their international counterparts. The mean scores for emotional exhaustion (35.5), depersonalization (14.66), and personal accomplishment (29.34) all exceed the threshold values established by Maslach et al. (32) for high burnout classification, indicating that the majority of Saudi community pharmacists experience significant burnout symptoms.

Sales pressure emerged as the strongest predictor of burnout, with pharmacists reporting constant sales pressure demonstrating significantly higher odds of experiencing emotional exhaustion (OR = 4.23) and depersonalization (OR = 3.45). The dose–response relationship observed between sales pressure frequency and burnout dimensions suggests a direct causal link between commercial pressures and psychological distress. This finding aligns with research by Jacobs et al. (8), who identified commercial pressures as significant contributors to workplace stress among community pharmacists in England, and highlights the tension between professional healthcare roles and commercial aspects of community pharmacy practice documented by Mott et al. (33).

The significant association between Saudi nationality and higher burnout scores (OR = 3.89 for emotional exhaustion, OR = 4.12 for depersonalization) represents a novel finding. This nationality-based difference may reflect cultural factors, differences in educational background, or varying expectations regarding work conditions and professional roles, as noted by Al-Arifi (17) regarding cultural factors affecting pharmacy practice in Saudi Arabia. Furthermore, this association between Saudi nationality and higher levels of emotional exhaustion and depersonalization may reflect distinct cultural and workplace dynamics. Saudi pharmacists may experience greater expectations for long-term career commitment, social responsibility, and family obligations, which can intensify work-related stress (34, 35). Cultural norms may discourage open discussion of mental health struggles, potentially limiting access to coping resources (36). These factors may amplify the psychological burden of professional demands. Similar nationality-based differences have been reported by Alameddine et al. (12) among community pharmacists in Lebanon, suggesting that cultural and contextual factors significantly influence burnout development across Middle Eastern pharmacy settings.

Professional experience serves as a significant protective factor against burnout, with each additional level of experience associated with reduced odds of emotional exhaustion (OR = 0.73) and depersonalization (OR = 0.78). This protective effect aligns with research by Gaither et al. (11) and suggests that targeted support for early-career pharmacists may be particularly important for burnout prevention. High patient volume emerged as a significant predictor of both emotional exhaustion (OR = 1.34) and depersonalization (OR = 1.41), consistent with findings from Çalgan et al. (9), reflecting the challenges of managing high workload while maintaining quality patient care.

A critical finding from our study is the differential pattern of predictors across the three burnout dimensions. While sales pressure, nationality, experience, and patient volume significantly predicted emotional exhaustion and depersonalization, personal accomplishment was primarily influenced by income level (OR = 1.34) and chain pharmacy employment (OR = 1.89). This distinct pattern supports the multidimensional conceptualization of burnout proposed by Maslach and Jackson (2), suggesting that different dimensions develop through distinct pathways and respond to different workplace factors. The finding that traditional stressors showed minimal association with personal accomplishment aligns with the findings of Schindel et al. (37), indicating that interventions targeting personal accomplishment may need to focus on professional development and recognition rather than workload reduction alone.

Overall, more recent studies consistently demonstrate that pharmacist burnout is a significant and growing concern worldwide, with moderate-to-high levels reported across diverse healthcare systems. In Saudi Arabia, research found that over half of pharmacists experienced clinically relevant burnout, with personal and work-related burnout rates exceeding 80% among community pharmacists (7, 38). These findings align with international studies that also report high rates of emotional exhaustion, depersonalization, and reduced professional efficacy among pharmacists, often linked to workload, administrative burden, and patient demands (3, 39). While these trends suggest that pharmacist burnout is a global phenomenon, unique contextual contributors in Saudi Arabia, such as rapid sector growth, evolving workforce demographics, workload expectations, communication styles, and limited support mechanisms, underscore the need for culturally sensitive interventions (38, 40).

### 4.1 Implications for practice and policy

The high prevalence of burnout and identified risk factors among Saudi community pharmacists have several important implications for pharmacy practice, education, and policy. First, the strong association between sales pressure and burnout suggests a need to reevaluate commercial performance metrics in community pharmacy settings and develop balanced approaches that recognize both professional care quality and business sustainability. Regulatory bodies and pharmacy organizations could develop guidelines for ethical business practices that minimize undue commercial pressure on pharmacy professionals. Second, the protective effect of experience highlights the importance of professional development and mentorship programs, particularly for early-career pharmacists. Structured transition-to-practice programs, mentorship initiatives, and enhanced professional support during the first years of practice could potentially reduce burnout risk during this vulnerable period.

Third, the nationality-based differences in burnout susceptibility suggest a need for culturally sensitive approaches to pharmacy education, practice standards, and workplace support. Educational institutions and regulatory bodies should consider cultural factors when developing pharmacy curricula, continuing education programs, and practice guidelines to ensure they address the specific challenges faced by Saudi pharmacists. Culturally sensitive strategies should be implemented at the workplace, including workplace wellness programs tailored to Saudi social values, structured peer-support or mentorship systems, and leadership training that promotes open communication and mental health awareness (34). Integrating these approaches into pharmacy practice could help reduce stigma, support emotional resilience, and improve retention among Saudi pharmacists (34, 35). Finally, the association between patient volume and burnout underscores the importance of adequate staffing levels, workflow optimization, and technological support in community pharmacy settings. Pharmacy owners, managers, and policymakers should prioritize staffing models that balance efficiency with pharmacist well-being to ensure sustainable practice environments.

Pharmacist burnout and mental well-being are critical issues in Saudi Arabia, with recent studies revealing high rates of burnout among pharmacy staff, particularly during the COVID-19 pandemic. For example, over half of pharmacists reported clinically significant burnout, with risk factors including younger age, female gender, fewer years of experience, high workload, lack of supportive work culture, and sleep disturbances (34, 38, 41). Emotional exhaustion and low personal accomplishment are especially prevalent among pharmacy faculty, influenced by factors such as citizenship, family responsibilities, and academic rank (34). Community pharmacists working longer hours or in smaller teams are at even greater risk, with personal and work-related burnout rates exceeding 80% (38). The pandemic has intensified these challenges, as fears of infection, increased working hours, and emotional distress have contributed to higher burnout levels (7, 42). Barriers to addressing mental health include stigma, lack of awareness, and insufficient access to mental health services, which are also reflected in the broader healthcare workforce and general population (43, 44). Effective interventions require institutional support, comprehensive training, and the integration of mental health promotion into workplace systems, aligning with both the United Nations Sustainable Development Goals and Saudi Arabia’s National Mental Health and Prevention Plan (45, 46). Addressing these issues is essential for sustaining a resilient healthcare workforce and improving overall healthcare quality.

### 4.2 Limitations

Several limitations should be considered when interpreting these findings. The cross-sectional design precludes causal inferences regarding the relationships between identified risk factors and burnout dimensions. The convenience sampling approach may have introduced selection bias, potentially limiting generalizability. Self-reported data are subject to recall bias and social desirability effects. The study did not explore potential mediating variables such as coping strategies or organizational culture. Finally, the study was conducted during a period of ongoing healthcare system transformation in Saudi Arabia, which may have influenced pharmacist experiences.

## 5 Conclusion

This study reveals alarmingly high prevalence rates of burnout among community pharmacists in Saudi Arabia, with approximately two-thirds of pharmacists experiencing high levels of emotional exhaustion, depersonalization, and reduced personal accomplishment. Sales pressure emerged as the strongest predictor of burnout, followed by Saudi nationality, high patient volume, and limited professional experience. The findings highlight the urgent need for targeted interventions to address burnout in this population, including reevaluation of commercial performance metrics, enhanced support for early-career pharmacists, culturally sensitive practice guidelines, and adequate staffing models. Addressing these factors is essential for promoting pharmacist well-being, ensuring quality patient care, and sustaining a resilient pharmacy workforce in Saudi Arabia.

## Data Availability

The raw data supporting the conclusions of this article will be made available by the authors upon request, without undue reservation.
